# Moral Courage Mediates the Relationship Between Ethical Climate and Sustainable Environmental Health Literacy Among Nurses

**DOI:** 10.3390/ijerph23050597

**Published:** 2026-05-01

**Authors:** Mirfat Mohamed Labib Elkashif, Mohamed Sayed Abdellatif, Darelglal Ahmed Gassmelseed Abdalla, Ashraf Ragab Ibrahim, Mohamed Ali Nemt-allah

**Affiliations:** 1 Department of Nursing Sciences, College of Applied Medical Sciences in Wadi Aldawaser, Prince Sattam Bin Abdulaziz University, Wadi Aldawaser 18616, Saudi Arabia; m.alkashif@psau.edu.sa (M.M.L.E.); d.elseed@psau.edu.sa (D.A.G.A.); 2 Department of Psychology, College of Education in Al-Kharj, Prince Sattam Bin Abdulaziz University, Al-Kharj 11942, Saudi Arabia; m.heby@psau.edu.sa; 3 Educational Psychology and Statistics Department, Faculty of Education, Al-Azhar University, Cairo 35822, Egypt; ashrafibrahem.26@azhar.edu.eg

**Keywords:** ethical climate, moral courage, environmental health literacy, sustainable healthcare, nursing practice, organizational culture

## Abstract

**Highlights:**

**Public health relevance—How does this work relate to a public health issue?**

- Climate change intensifies health burdens globally, yet healthcare systems contribute substantially to greenhouse gas emissions; nurses, as the largest health workforce, are uniquely positioned to address this dual challenge through sustainable environmental health literacy.
- Organizational ethical climate shapes nurses’ capacity to access, understand, and apply environmental health information, creating direct implications for climate-responsive care delivery and healthcare system decarbonization.

**Public health significance—Why is this work of significance to public health?**

- This study provides empirical evidence that moral courage statistically associates as a partial mediator of the relationship between ethical climate and sustainable environmental health literacy among nurses, extending existing mediation frameworks to the environmental health domain.
- Supportive ethical climates explain 74% of variance in nurses’ environmental health literacy and 66% of variance in moral courage, demonstrating that institutional culture is a powerful, modifiable determinant of climate-responsive nursing practice.

**Public health implications—What are the key implications or messages for practitioners, policy makers and/or researchers in public health?**

- Healthcare administrators and policymakers should prioritize building ethical organizational climates characterized by psychological safety, participatory decision-making, and explicit sustainability values to equip nurses with both the knowledge and courage needed for sustainable practice.
- Nursing education and professional codes of conduct must integrate planetary health imperatives and moral courage development alongside clinical training, legitimizing environmental stewardship as a core professional responsibility essential for climate-resilient healthcare transformation.

**Abstract:**

Despite growing recognition that organizational culture shapes nursing practice, the linking of ethical climate to sustainable environmental health literacy (SEHL) remains poorly understood. This study examined whether moral courage statistically mediates the relationship between perceived ethical climate and self-reported environmental health literacy among Egyptian nurses, rather than observed competencies or clinical actions. A cross-sectional correlational design was employed with 743 nurses recruited from government, private, and university-affiliated hospitals. Participants completed the Hospital Ethical Climate Survey, the Nurses’ Moral Courage Scale, and the Environmental Health Literacy Scale. Mediation analysis used Hayes’ PROCESS macro with 5000 bootstrap samples. Ethical climate demonstrated strong positive associations with moral courage (r = 0.81) and SEHL (r = 0.86), while moral courage and SEHL were also strongly correlated (r = 0.82). Ethical climate explained 74% of variance in SEHL and 66% of variance in moral courage. Moral courage was associated with partial statistical mediation of the ethical climate–SEHL relationship, accounting for 33.4% of the total effect (β = 0.31, 95% CI [0.26, 0.37]), while the direct effect remained substantial (66.6%). These findings suggest that supportive ethical climates may be associated with nurses’ environmental health literacy via two statistical pathways: one directly linked to environmental learning and another indirectly linked to moral courage. Healthcare organizations should prioritize ethical climate development alongside moral courage training as potentially promising approaches to advance climate-responsive nursing practice.

## 1. Introduction

The nursing profession confronts a fundamental ethical tension between traditional patient-centered care principles and emerging planetary health imperatives. While conventional nursing ethics prioritizes immediate individual patient welfare, planetary health ethics extends professional obligations to encompass populations, ecosystems, and future generations [1,2]. This conflict becomes particularly acute in clinical settings where institutional norms mandate resource-intensive, disposable-heavy practices framed as essential for patient safety and efficiency, directly opposing nurses’ growing responsibility to practice sustainably [3,4,5]. Critical care environments exemplify this dilemma, generating substantial waste and carbon footprints daily while operating under performance pressures that deprioritize environmental stewardship [6,7]. Consequently, nurses find themselves navigating competing ethical demands: the immediate “do no harm” mandate to present patients versus the collective duty to mitigate long-term environmental harm [8,9,10].

Sustainable Environmental Health Literacy (SEHL) has emerged as an essential competency for nurses positioned at the critical intersection of healthcare delivery and climate change. Climate change intensifies health burdens through heat-related illnesses, respiratory diseases, cardiovascular complications, and disaster-related injuries, while healthcare systems themselves contribute approximately 1–5% of global greenhouse gas emissions [11,12]. As the largest and most trusted health workforce, nurses serve as frontline advocates capable of addressing this dual challenge [1,13,14]. Evidence demonstrates that SEHL enables nurses to deliver climate-responsive clinical care, lead decarbonization initiatives, advocate for climate justice, and drive cultural transformation toward sustainable practice [6,15,16,17]. Furthermore, educational interventions integrating planetary health concepts significantly enhance nurses’ environmental awareness, self-efficacy, and pro-environmental behaviors essential for building climate-resilient healthcare systems [18,19,20].

Ethical climate operates as a critical meso-level organizational feature situated between individual nurse values and broader health system structures, fundamentally shaping professional behavior through shared perceptions of ethical procedures and power dynamics [21,22]. Manifested through both formal mechanisms—including leadership practices, decision-making processes, and reward systems—and informal norms such as role modeling and organizational discourse, ethical climate directly influences nurses’ caring behaviors, organizational citizenship, and ethical decision-making capacity [23,24]. Positive ethical climates characterized by fair power distribution and inclusive ethical procedures foster trust, job satisfaction, professional commitment, and retention [25,26]. Conversely, climates marked by hierarchical rigidity, favoritism, or silencing of ethical concerns generate moral distress and turnover intentions [27,28,29]. Consequently, ethical climate shapes the organizational conditions through which institutional power structures either enable or constrain nurses’ capacity to enact professional values [30,31].

The absence of moral courage in nursing practice negatively affects multiple stakeholder groups, with patients bearing the most immediate burden through compromised safety and care quality [32,33,34]. Diminished moral courage correlates with increased patient safety silence, reduced error reporting, and failures to advocate for safe practices, ultimately undermining public health outcomes and system-level safety culture [30,35,36]. Critically, nurses themselves experience profound harm when institutional barriers—including hierarchical constraints, fear of retaliation, and disempowerment—prevent them from acting on ethical knowledge [37,38,39]. This suppression generates moral distress, burnout, and professional disengagement while specifically inhibiting nurses’ capacity to address environmental and sustainability concerns despite possessing relevant knowledge [40,41,42]. Consequently, inadequate moral courage perpetuates a cycle wherein nurses remain silent on unsafe or unsustainable practices, thereby compromising both patient welfare and planetary health imperatives [43,44].

Ethical climate may influence nurses’ environmental literacy through a motivational pathway wherein supportive organizational environments provide the conditions—including psychological safety, normative support, and access to resources—that facilitate learning and sustainable practice adoption. Positive ethical climates characterized by fair leadership, participatory decision-making, and explicit sustainability values enhance intrinsic motivation and professional engagement, thereby encouraging nurses to pursue climate-related knowledge and competencies [45,46,47]. When organizations frame planetary health as integral to professional ethics rather than optional, nurses develop stronger environmental stewardship identities and increased willingness to engage with sustainability science [2,3,15]. Evidence demonstrates that supportive work environments enabling climate activism and innovation significantly enhance nurses’ environmental self-efficacy, climate literacy, and green work behaviors including waste reduction and resource conservation [17,18]. Consequently, ethical climate may translate organizational values into environmental literacy and sustainable clinical practices [48,49].

Moral courage may function as a critical mediating mechanism statistically bridging the persistent knowledge-action gap between environmental awareness and sustainable practice implementation. While positive ethical climates strengthen moral awareness and environmental literacy enhances understanding of sustainability imperatives, these factors alone insufficiently translate into behavioral change when action involves personal risk, institutional conflict, or professional sacrifice [50,51]. Research consistently demonstrates that environmental knowledge increases concern and perceived responsibility, yet intentions only partially convert to behavior without psychological catalysts that enable courage-like action [52,53]. Moral courage may function precisely at this tension point, potentially empowering nurses to act on ethical judgments despite fear of negative consequences, functioning analogously to moral obligation and responsibility constructs that mediate knowledge-behavior relationships in sustainability contexts [30,54,55]. Accordingly, moral courage may be associated with transforming informed environmental concern into concrete pro-environmental behaviors when organizational pressures and systemic barriers would otherwise inhibit action [56].

This study integrates Social Cognitive Theory and Rest’s Model of Moral Action to explain how organizational ethical climate—operationalized here via the Hospital Ethical Climate Survey (HEC)—translates into SEHL—measured by the Environmental Health Literacy Scale (EHL)—through moral courage—assessed using the Nurses’ Moral Courage Scale (NMC). Social Cognitive Theory elucidates the reciprocal determinism whereby ethical climates shape nurses’ attitudes, self-efficacy, and outcome expectations regarding sustainable practices through role modeling, normative cues, and reinforcement mechanisms [57,58,59,60]. Specifically, HEC scores capture these normative and relational dimensions, representing the organizational inputs that, per Social Cognitive Theory, condition individual competency development.

Simultaneously, Rest’s Model delineates the internal moral journey from sensitivity to environmental harms, through ethical judgment and motivation, culminating in moral courage to act despite institutional barriers [34,61,62,63]. NMC scores operationalize this moral action stage, functioning as the volitional bridge between organizational conditions and environmental practice. This integrated framework demonstrates how supportive ethical climates cultivate sustainable health literacy—the capacity to obtain, appraise, and apply environmental health information—while simultaneously strengthening moral courage necessary for translating organizational sustainability values into actual clinical practice [55,64,65]. Accordingly, the mediation model tested here positions HEC as the independent variable, NMC as the mediator, and EHL as the outcome, directly reflecting the theoretical sequence from organizational climate through moral action to sustainability competence.

Despite growing recognition of each component’s importance, empirical integration of these constructs remains absent. While ethical climate predicts professional behavior and moral courage mediates knowledge-action gaps, no quantitative study has examined moral courage as a mediator linking ethical climate to environmental health literacy in nursing. Moral courage scholarship identifies antecedents and leadership relationships without connecting findings to environmental practice [30,34,41]. Sustainability research targets green competence and climate education without incorporating ethical climate or moral courage as explanatory mechanisms [48,66,67]. This leaves a specific empirical gap: the organizational and psychological pathways through which nurses develop environmental health literacy remain untested. This study addresses this gap by testing a mediation model positioning moral courage as the mechanism linking ethical climate to sustainable environmental health literacy.

This study aimed to examine self-reported perceptions of ethical climate, moral courage, and SEHL in Egyptian nurses while testing a mediation model. Specifically, the study sought to determine whether positive ethical climates directly enhance nurses’ capacity to access, understand, verify, and apply environmental health information, and whether this relationship operates indirectly through strengthening nurses’ moral courage to advocate for sustainable practices. By investigating both direct and indirect pathways, this research contributes to understanding how organizational contexts shape nurses’ environmental competencies and ethical action capacities, extending established mediation frameworks to the underexplored domain of environmental health literacy. The study hypothesized that ethical climate would demonstrate significant positive associations with both moral courage and environmental health literacy, that moral courage would positively predict environmental health literacy, and that moral courage would partially mediate the relationship between ethical climate and environmental health literacy, thereby explaining how organizational environments translate into individual-level sustainability competencies and behaviors.

## 2. Materials and Methods

### 2.1. Study Design

This cross-sectional correlational study employed a quantitative research design to examine the mediating role of moral courage in the relationship between ethical climate and SEHL among Egyptian nurses. Data were collected through structured self-report questionnaires administered between 29 September and 25 October 2025. The study utilized a two-phase approach: an initial psychometric validation phase followed by the main data collection phase to test the proposed mediation model.

### 2.2. Participants and Sampling

The study comprised two distinct samples serving different analytical purposes. The psychometric validation sample (Phase 1) was recruited first to establish Arabic-adapted scale adequacy within the Egyptian nursing context before main data collection commenced. This sample consisted of 534 Egyptian nurses aged 22–59 years (M = 34.85, SD = 7.71) with professional experience ranging from 1–19 years (M = 5.83, SD = 3.77). The main study sample (Phase 2) included 743 Egyptian nurses aged 23–60 years (M = 35.57, SD = 8.27) with experience ranging from 1–19 years (M = 5.41, SD = 3.49). Both samples were independent; no participant contributed data to both phases.

Participants were recruited using convenience sampling from government, private, and university-affiliated hospitals across Cairo, and Dakahlia governorates. Recruitment proceeded through direct approach by research team members during nursing staff meetings and ward rounds. Recruitment sites were selected to capture variability across hospital types and clinical contexts representative of the Egyptian healthcare system, though purposive representation of rural or under-resourced facilities was not achieved. Of 900 questionnaires distributed in Phase 2, 743 were returned and deemed eligible for analysis, yielding a response rate of 82.6%. Incomplete questionnaires (*n* = 23) were excluded; remaining non-returns (*n* = 134) were not followed up systematically. Reasons for non-participation were not formally recorded, though informal feedback suggested workload and time constraints as primary factors. Detailed demographic characteristics of both samples are presented in Table 1.

Inclusion criteria required participants to be registered nurses actively employed in direct patient care roles with a minimum of one year of clinical experience. Nurses in administrative positions without direct patient contact were excluded. These criteria ensured respondents possessed sufficient organizational exposure to meaningfully perceive ethical climate dimensions and adequate clinical experience to contextualize environmental health decisions within practice.

### 2.3. Measures

The Hospital Ethical Climate Survey (HEC) was employed to assess nurses’ perceptions of the ethical climate within their healthcare institution [68]. This 26-item instrument measures five dimensions reflecting fundamental workplace relationships: peers, patients, managers, hospital/organization, and physicians. Respondents rated each item using a 5-point Likert scale ranging from 1 (*almost never true*) to 5 (*almost always true*), with higher scores indicating more positive ethical climate perceptions. Confirmatory factor analysis demonstrated excellent model fit (CMIN/DF = 1.15, GFI = 0.95, CFI = 0.98, RMSEA = 0.01). Within the current sample, convergent validity was supported by strong factor loadings across all five dimensions, and discriminant validity was evidenced by moderate inter-dimension correlations (r = 0.45–0.58), confirming that dimensions capture related yet distinct aspects of ethical climate. Internal consistency reliability was strong across all dimensions, with omega coefficients ranging from 0.77 (peers) to 0.83 (physicians), and an overall scale omega of 0.84. Inter-dimension correlations (r = 0.53–0.62) remained moderate, supporting discriminant validity across the five relational dimensions despite shared organizational context.

The Nurses’ Moral Courage Scale (NMC) was utilized to measure self-perceived moral courage in nursing practice [69]. This 21-item instrument comprises four dimensions: compassion and true presence (CTP), moral responsibility (MR), moral integrity (MI), and commitment to good care (CGC). Items were rated on a 5-point Likert scale from 1 (does not describe me at all) to 5 (describes me very well), with higher scores reflecting greater moral courage. The scale demonstrated satisfactory model fit indices (CMIN/DF = 1.21, GFI = 0.96, CFI = 0.98, RMSEA = 0.02). Construct validity within the present sample was supported by acceptable convergent validity, with all subscales correlating strongly with the total score (r = 0.74–0.83), while inter-dimension correlations remained moderate (r = 0.48–0.59), indicating sufficient discriminant validity among moral courage facets. Reliability analysis revealed acceptable to excellent internal consistency, with omega coefficients ranging from 0.78 (CTP) to 0.85 (MI), and a total scale omega of 0.86.

The Environmental Health Literacy Scale (EHL) was administered to evaluate nurses’ knowledge and skills related to environmental health [70]. This 25-item measure assesses four dimensions: access to environmental health information (AEHI), understanding of environmental health information (UEHI), verification of environmental health information (VEHI), and health-protective decision-making (HPDM). Participants responded using a 5-point Likert scale ranging from 1 (not at all true of me) to 5 (completely true of me), with higher scores indicating greater environmental health literacy. The instrument exhibited excellent psychometric properties with strong model fit (CMIN/DF = 1.07, GFI = 0.96, CFI = 0.99, RMSEA = 0.01). Construct validity was demonstrated through strong subscale-to-total correlations (r = 0.80–0.88) supporting convergent validity, alongside moderate inter-subscale correlations (r = 0.52–0.71) confirming discriminant validity across literacy dimensions. Internal consistency was high across all subscales, with omega coefficients ranging from 0.83 (HPDM) to 0.87 (AEHI), and an overall omega of 0.89.

### 2.4. Translation and Adaptation Procedure

All three instruments were translated into Arabic using a forward-backward translation protocol. Two bilingual nursing academics independently produced forward translations, which were reconciled by consensus. Two different translators independently back-translated the reconciled version, and discrepancies were resolved through expert panel review including three nursing faculty and one clinical nurse specialist. Minor linguistic adaptations were made to ensure cultural appropriateness within the Egyptian healthcare context; no items were deleted or structurally modified. The adapted Arabic versions were piloted with 20 nurses prior to Phase 1 data collection to confirm item clarity.

CFA was conducted on all three instruments within the current sample rather than relying solely on original validations because prior validated versions were developed in non-Arabic, non-Egyptian contexts. Local psychometric verification was therefore necessary to confirm structural validity before proceeding to hypothesis testing.

### 2.5. Data Analysis

Data were analyzed using SPSS 27 and AMOS 26. Preliminary analyses included descriptive statistics, normality assessment, and confirmatory factor analysis to validate measurement models. To address potential multicollinearity concerns arising from strong inter-construct correlations, variance inflation factors (VIF) were examined for all predictors in the mediation model. All VIF values fell below 3.0, indicating acceptable multicollinearity levels and confirming that ethical climate and moral courage, despite their strong correlation (r = 0.81), contribute distinct predictive variance to environmental health literacy. Pearson correlations examined bivariate relationships among variables. Given the exploratory and confirmatory nature of the hypothesized relationships grounded in established theory, adjustments for multiple comparisons were not applied; however, we acknowledge this increases Type I error risk and readers should interpret correlation significance values conservatively.

Common method variance was assessed using Harman’s single-factor test, which indicated that common method bias was not a concern (18.41% variance explained). The mediation analysis employed PROCESS macro Model 4 [71] (with 5000 bootstrap samples to generate bias-corrected 95% confidence intervals for indirect effects. PROCESS macro was selected as the primary analytical approach given its bootstrapping capability for indirect effect estimation; while structural equation modeling would simultaneously estimate measurement and structural parameters, the current sample size and cross-sectional design supported PROCESS as an appropriately rigorous and parsimonious choice for testing the single-mediator model.

## 3. Results

Descriptive analyses revealed variability across all measured constructs and their constituent dimensions. Table 2 presents the means, standard deviations, and ranges for all study variables. Ethical climate subscales demonstrated moderate to moderately high mean scores, with physician relationships showing the highest average and patients relationships showing the lowest. Moral courage dimensions indicated that MI displayed the highest mean, while MR showed the lowest. For environmental health literacy, AEHI yielded the highest mean, whereas HPDM produced the lowest. These descriptive patterns suggest that while nurses generally perceive moderate ethical climates and demonstrate reasonable moral courage and environmental health literacy, substantial room for improvement exists across all domains. Specifically, all three total scale means exceeded their respective theoretical midpoints (ethical climate: 78; moral courage: 52.5; EHL: 75), yet remained well below ceiling, indicating moderate rather than strong levels across constructs. The lowest subscale means—patient relationships (M = 11.36), moral responsibility (M = 10.79), and health-protective decision-making (M = 15.31)—identify the most actionable areas for intervention.

Bivariate correlation analyses examining relationships among all study variables are displayed in Table 3. All correlations were positive and statistically significant at the *p* < 0.01 level, indicating strong interconnections across ethical climate, moral courage, and environmental health literacy constructs. Within ethical climate dimensions, correlations ranged from moderate to strong (r = 0.47 to 0.58). Moral courage dimensions exhibited moderate to strong intercorrelations (r = 0.47 to 0.54), with all subscales strongly correlating with total moral courage scores (r = 0.74 to 0.83). Environmental health literacy dimensions showed moderate to strong intercorrelations (r = 0.571 to 0.691), with particularly high correlations between subscales and total scores (r = 0.80 to 0.88). Critically, ethical climate demonstrated strong positive correlations with both moral courage (r = 0.81) and environmental health literacy (r = 0.86), while moral courage and environmental health literacy were also strongly correlated (r = 0.82). These correlation patterns provide preliminary support for the hypothesized relationships among study constructs.

The mediation model examining moral courage as a mediator of the relationship between ethical climate and environmental health literacy was tested using Hayes’ PROCESS macro with 5000 bootstrap samples. Results are presented in Table 4 and Table 5.

The total effect of ethical climate on environmental health literacy was statistically significant (β = 0.94, *p* < 0.001), with ethical climate explaining 74% of the variance. While this exceeds conventional large effect benchmarks, the magnitude likely reflects partial inflation from shared method variance and conceptual proximity among self-reported constructs, and should not be interpreted as indicating equivalent predictive utility in objective behavioral outcomes. When moral courage was introduced as a mediator, ethical climate accounted for 66% of its variance. Both ethical climate (β = 0.62, *p* < 0.001) and moral courage (β = 0.51, *p* < 0.001) significantly predicted environmental health literacy, together explaining 78% of variance. The indirect effect was statistically significant (β = 0.31, 95% CI [0.26, 0.37]), accounting for 33.4% of the total effect. This indirect magnitude indicates that moral courage contributes meaningfully beyond ethical climate alone, supporting it as a complementary rather than redundant intervention target.

The decomposition of effects revealed that ethical climate exerts both direct and indirect influences on environmental health literacy, with the direct pathway remaining substantial even after accounting for moral courage. Table 5 summarizes the total, direct, and indirect effects alongside their respective contributions to the overall relationship.

**Figure 1:**
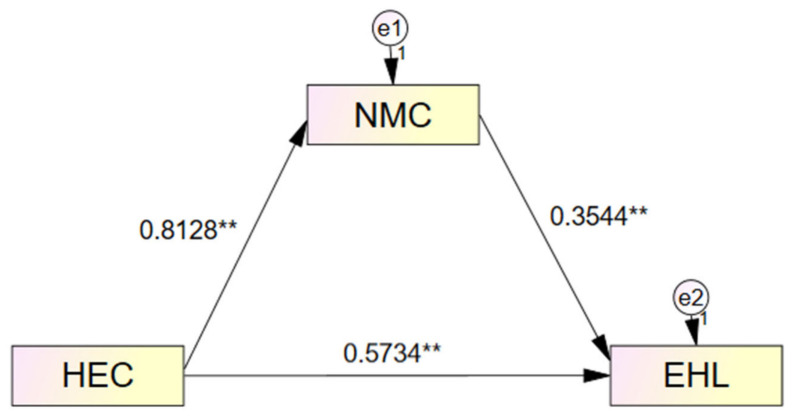
Mediation Model Showing Standardized Path Coefficients for the Relationship Between Ethical Climate and Environmental Health Literacy Through Moral Courage. Note. ** *p* < 0.01.

Figure 1 illustrates the mediation model with standardized path coefficients. The model demonstrates that ethical climate has a strong direct effect on moral courage (β = 0.81, *p* < 0.01), which in turn significantly influences environmental health literacy (β = 0.35, *p* < 0.01). The direct path from ethical climate to environmental health literacy remains substantial (β = 0.57, *p* < 0.01) even after controlling for moral courage, confirming partial mediation. The total effect of ethical climate on environmental health literacy (β = 0.86, *p* < 0.01) is decomposed into a direct effect accounting for 66.6% and an indirect effect through moral courage accounting for 33.4% of the total influence.

The results provide robust empirical support for the hypothesized mediation model. First, ethical climate, moral courage, and environmental health literacy demonstrated strong positive intercorrelations, with coefficients ranging from 0.61 to 0.86 for primary constructs. Second, ethical climate significantly predicted both moral courage and environmental health literacy, explaining 66% and 74% of their respective variances. Third, moral courage partially mediated the relationship between ethical climate and environmental health literacy, accounting for one-third of the total effect while the direct pathway remained significant. These findings suggest perceived ethical climates may be associated with nurses’ self-reported environmental health literacy via two statistical pathways: one directly linked to reported environmental learning and another indirectly linked through self-assessed moral courage.

## 4. Discussion

The current study provides associational evidence suggesting that ethical climate is linked to both direct and indirect statistical associations with nurses’ environmental health literacy, with moral courage serving as a significant partial mediator accounting for one-third of the total statistical effect. This two-pathway association reveals that supportive organizational ethical climates may simultaneously be linked to nurses’ capacity to access, understand, and apply environmental health information while potentially cultivating moral courage. Examination of subscale patterns provides additional analytical insight: among ethical climate dimensions, physician relationships yielded the highest mean while patients relationships scored lowest, suggesting nurses perceive stronger ethical alignment with physician colleagues than with patient-centered ethical procedures, potentially reflecting hierarchical dynamics within Egyptian hospital contexts. While the high explained variance proportions (74%, 66%) reflect strong organizational associations consistent with theory, they may be partially inflated by shared method variance and conceptual proximity among constructs; given the cross-sectional self-report design, these figures should be interpreted cautiously and true population effects are likely more modest.

The substantial direct effect (66.6%) suggests that ethical climates create enabling conditions—including psychological safety, resource availability, and institutional legitimacy—that facilitate environmental learning independent of individual courage. Conversely, the meaningful indirect pathway (33.4%) indicates that ethical climates also strengthen nurses’ willingness to challenge unsustainable practices and advocate for planetary health despite institutional resistance. Within moral courage dimensions, moral integrity scored highest while moral responsibility scored lowest, suggesting nurses recognize ethical obligations cognitively but experience greater difficulty assuming personal accountability for action—a distinction with direct implications for targeted intervention design. Furthermore, among EHL dimensions, access to environmental health information yielded the highest mean whereas health-protective decision-making scored lowest, reinforcing the knowledge-action gap that moral courage theoretically bridges and empirically partially explains in the current mediation model. These findings illuminate how organizational culture shapes both cognitive competencies and volitional capacities essential for sustainable healthcare transformation.

These findings are broadly consistent with established literature demonstrating that positive ethical climates are associated with trust, professional commitment, and ethical decision-making capacity, while extending this associational framework to encompass environmental stewardship. The strong inter-construct correlations (r = 0.81–0.86), while theoretically expected given shared professional values foundations, raise potential conceptual overlap concerns. However, CFA-confirmed discriminant validity and VIF values below 3.0 collectively support that constructs remain sufficiently distinct for mediation analysis. The strong relationship between ethical climate and environmental health literacy aligns with evidence that supportive work environments significantly enhance nurses’ climate literacy and green behaviors [17,18]. The statistical mediating role of moral courage aligns with research identifying it as potentially critical for bridging knowledge-action gaps when behavior involves institutional conflict [30,55]. Furthermore, results support theoretical propositions that ethical climates function as motivational catalysts stimulating sustainability learning [45,47], while moral courage enables translation of environmental concern into pro-environmental action despite barriers [54]. This integrated model addresses identified gaps by simultaneously embedding ethical principles, operationalizing moral courage, and targeting sustainable practices within organizational contexts.

These associational findings suggest several potentially promising directions, though causal conclusions cannot be drawn and recommendations should be interpreted cautiously pending experimental verification. Should longitudinal studies confirm these associations, healthcare administrators might consider cultivating ethical climates characterized by participatory decision-making, psychological safety, and transparent sustainability values, as these organizational features showed the strongest statistical associations with nurses’ environmental competencies. Educational programs may benefit from incorporating moral courage development alongside environmental literacy training, given moral courage’s partial mediating role in this sample. At a broader level, embedding planetary health perspectives within professional nursing frameworks represents a plausible—though empirically unconfirmed—direction for supporting climate-responsive practice. Specific mechanisms such as sustainability committees or environmental performance metrics remain speculative intervention targets suggested by these associations rather than evidence-based prescriptions. Stronger experimental and longitudinal designs are needed before concrete institutional or policy recommendations can be responsibly advanced.

Several limitations warrant consideration. First, the cross-sectional design precludes causal inferences, as reciprocal relationships may exist wherein environmental literacy shapes ethical climates or moral courage influences organizational ethics perceptions. Second, convenience sampling limits generalizability; participants were drawn exclusively from accessible Egyptian hospitals, potentially overrepresenting better-resourced institutions, and findings should not be extrapolated to rural, under-resourced, or culturally different healthcare settings. The contextual specificity of the Egyptian healthcare environment—characterized by hierarchical structures, resource constraints, and cultural professional norms—further limits transferability to Western or high-income healthcare systems where ethical climate manifestations and sustainability priorities differ substantially. Third, self-report measures introduce social desirability bias, particularly for moral courage, where participants may overestimate courageous behavior in hypothetical versus real high-stakes situations. Beyond common method variance assessment, HEC, NMC, and EHL scores reflect perceived rather than objectively verified behaviors and organizational conditions, potentially inflating inter-construct correlations. Common method bias is a substantial limitation. All constructs were self-reported simultaneously using identical formats, and Harman’s single-factor test—the only bias assessment employed—lacks sensitivity to confirm bias absence. The high inter-construct correlations (r = 0.81–0.86) are consistent with method-induced inflation.

Future investigations should employ experimental designs testing targeted interventions that manipulate ethical climate dimensions to establish causal pathways and identify which specific organizational features most powerfully enhance environmental literacy and moral courage. Longitudinal studies tracking nurses across career stages could illuminate developmental trajectories, examining whether moral courage precedes environmental literacy acquisition or emerges as consequence of sustainability knowledge combined with supportive climates. Qualitative research exploring nurses’ lived experiences navigating ethical dilemmas between patient safety protocols and environmental sustainability would provide rich contextual understanding of courage manifestation in practice. Cross-cultural comparative studies examining model validity across healthcare systems with varying environmental regulations and professional autonomy levels would enhance theoretical generalizability. Finally, multilevel modeling incorporating unit-level climate assessments, individual nurse characteristics, and patient outcomes could reveal how organizational ethical climates cascade through moral courage and environmental literacy to ultimately impact care quality and ecological footprints.

## 5. Conclusions

This study establishes associational evidence linking self-reported perceptions of organizational ethical climate to self-reported SEHL, with all constructs reflecting perceived rather than observed nursing practice. These findings suggest that ethical work environments may directly facilitate environmental learning while also strengthening moral courage to enact sustainable practices. The partial mediation model suggests organizational interventions may need to address both structural enablers of environmental literacy and psychological empowerment for courageous action. Given the cross-sectional self-report design with convenience sampling, causal conclusions cannot be drawn, and these findings should be regarded as preliminary and hypothesis-generating. Successfully integrating ethical climate development, moral courage cultivation, and environmental literacy enhancement represents a potentially promising pathway toward climate-resilient nursing practice.

## Figures and Tables

**Table 1:** Demographic Characteristics of Psychometric and Main Samples.

Variable	Category	Psychometric Sample		Main Sample	
		*N*	%	*N*	%
Gender	Male	92	17.2	121	16.3
	Female	442	82.8	622	83.7
Education	Diploma	81	15.2	101	13.6
	Bachelor	342	64.0	505	68.0
	Master	99	18.5	118	15.9
	PhD	12	2.2	19	2.6
Hospital type	Government	238	44.6	368	49.5
	Private	197	36.9	247	33.2
	University	99	18.5	128	17.2
Department	ICU	112	21.0	126	17.0
	ER	152	28.5	156	21.0
	Surgery	92	17.2	152	20.5
	Internal	110	20.6	159	21.4
	Pediatrics	68	12.7	150	20.2

**Table 2:** Descriptive Statistics for Study Variables.

Variable	Minimum	Maximum	Mean	SD
Peers	4.00	18.00	11.83	2.62
Patients	4.00	18.00	11.36	2.44
Managers	6.00	24.00	16.65	3.35
Hospital	6.00	24.00	16.18	3.17
Physicians	6.00	26.00	16.94	3.38
Ethical Climate	26.00	89.00	72.98	11.85
CTP	5.00	21.00	13.24	2.79
MR	4.00	16.00	10.79	2.32
MI	7.00	27.00	17.94	3.57
CGC	5.00	20.00	13.38	2.73
Moral Courage	21.00	72.00	55.36	9.02
AEHI	7.00	31.00	23.01	4.48
UEHI	7.00	30.00	21.04	4.05
VEHI	6.00	26.00	17.57	3.59
HPDM	5.00	22.00	15.31	3.14
EHL	25.00	94.00	76.94	12.88

**Table 3:** Pearson Correlation Coefficients Among Ethical Climate, Moral Courage, and Environmental Health Literacy Variables.

Variable	1	2	3	4	5	6	7	8	9	10	11	12	13	14	15	16
1. Peers	1															
2. Patients	0.45 **	1														
3. Managers	0.51 **	0.50 **	1													
4. Hospital	0.55 **	0.52 **	0.53 **	1												
5. Physicians	0.52 **	0.51 **	0.57 **	0.58 **	1											
6. Ethical Climate	0.76 **	0.74 **	0.80 **	0.81 **	0.82 **	1										
7. CTP	0.47 **	0.46 **	0.53 **	0.51 **	0.51 **	0.63 **	1									
8. MR	0.45 **	0.47 **	0.50 **	0.54 **	0.51 **	0.63 **	0.47 **	1								
9. MI	0.50 **	0.49 **	0.52 **	0.53 **	0.52 **	0.65 **	0.48 **	0.48 **	1							
10. CGC	0.51 **	0.50 **	0.53 **	0.54 **	0.52 **	0.66 **	0.49 **	0.49 **	0.54 **	1						
11. Moral Courage	0.61 **	0.61 **	0.66 **	0.67 **	0.65 **	0.81 **	0.77 **	0.74 **	0.83 **	0.80 **	1					
12. AEHI	0.56 **	0.58 **	0.61 **	0.62 **	0.62 **	0.76 **	0.59 **	0.58 **	0.58 **	0.57 **	0.73 **	1				
13. UEHI	0.59 **	0.58 **	0.58 **	0.60 **	0.60 **	0.75 **	0.57 **	0.56 **	0.55 **	0.60 **	0.72 **	0.69 **	1			
14. VEHI	0.52 **	0.51 **	0.55 **	0.56 **	0.55 **	0.68 **	0.50 **	0.49 **	0.53 **	0.50 **	0.64 **	0.60 **	0.60 **	1		
15. HPDM	0.55 **	0.52 **	0.56 **	0.59 **	0.56 **	0.71 **	0.50 **	0.51 **	0.54 **	0.52 **	0.66 **	0.60 **	0.59 **	0.57 **	1	
16. EHL	0.66 **	0.66 **	0.69 **	0.70 **	0.69 **	0.86 **	0.64 **	0.64 **	0.66 **	0.65 **	0.82 **	0.88 **	0.87 **	0.81 **	0.80 **	1

Note. ** *p* < 0.01.

**Table 4:** Unstandardized and Standardized Path Coefficients for the Mediation Model Testing Moral Courage as Mediator Between Ethical Climate and Environmental Health Literacy.

Path	β (Unstandardized)	SE	t	*p*	95% CI	Standardized β	R^2^
Path a: HEC → NMC	0.61	0.01	37.98	<0.01	[0.58, 0.65]	0.81	0.66
Path b: NMC → EHL	0.50	0.04	12.10	<0.01	[0.42, 0.58]	0.35	—
Path c’: HEC → EHL	0.62	0.03	19.58	<0.01	[0.56, 0.68]	0.57	0.78
Path c: HEC → EHL (total)	0.93	0.02	46.18	<0.01	[0.89, 0.97]	0.86	0.74

Note. Bootstrap samples = 5000. Confidence intervals are bias-corrected.

**Table 5:** Total, Direct, and Indirect Effects of Ethical Climate on Environmental Health Literacy with Percentage Contributions.

Effect Type	Unstandardized Effect	SE	95% CI	Standardized Effect	% of Total Effect
Total Effect	0.93	0.02	[0.89, 0.97]	0.86	100.0%
Direct Effect	0.62	0.03	[0.56, 0.68]	0.57	66.6%
Indirect Effect (via NMC)	0.31	0.02	[0.26, 0.36]	0.28	33.4%

## Data Availability

The datasets generated and analyzed during the current study are available from the corresponding author upon reasonable request.

## Abbreviations

The following abbreviations are used in this manuscript:

HEC	Hospital Ethical Climate
NMC	Nurses’ Moral Courage
EHL	Environmental Health Literacy
SEHL	Sustainable Environmental Health Literacy
CTP	Compassion and True Presence
MR	Moral Responsibility
MI	Moral Integrity
CGC	Commitment to Good Care
AEHI	Access to Environmental Health Information
UEHI	Understanding of Environmental Health Information
VEHI	Verification of Environmental Health Information
HPDM	Health-Protective Decision-Making
CI	Confidence Interval
